# Intracellular tracing of amyloid vaccines through direct fluorescent labelling

**DOI:** 10.1038/s41598-018-20845-9

**Published:** 2018-02-05

**Authors:** Matthew Mold, Manpreet Kumar, Ambreen Mirza, Emma Shardlow, Christopher Exley

**Affiliations:** The Birchall Centre, Lennard-Jones Laboratories, Keele University, Keele, Staffordshire ST5 5BG UK

## Abstract

Alzheimer’s disease is a debilitating neurodegenerative condition that progressively causes synaptic loss and major neuronal damage. Immunotherapy utilising Aβ as an active immunogen or via passive treatment utilising antibodies raised to amyloid have shown therapeutic promise. The migratory properties of peripheral blood-borne monocytes and their ability to enter the central nervous system, suggests a beneficial role in mediating tissue damage and neuroinflammation. However, the intrinsic phagocytic properties of such cells have pre-disposed them to internalise misfolded amyloidogenic peptides that could act as seeds capable of nucleating amyloid formation in the brain. Mechanisms governing the cellular fate of amyloid therefore, may prove to be key in the development of future vaccination regimes. Herein, we have developed unequivocal and direct conformation-sensitive fluorescent molecular probes that reveal the intracytoplasmic and intranuclear persistence of amyloid in a monocytic T helper 1 (THP-1) cell line. Use of the pathogenic Aβ_42_ species as a model antigen in simulated vaccine formulations suggested differing mechanisms of cellular internalisation, in which fibrillar amyloid evaded lysosomal capture, even when co-deposited on particulate adjuvant materials. Taken collectively, direct fluorescent labelling of antigen-adjuvant complexes may serve as critical tools in understanding subsequent immunopotentiation in vaccines directed against amyloidosis and wider dementia.

## Introduction

Alzheimer’s disease (AD) is the most prevalent form of dementia manifesting neurologically and symptomatically arising through the loss of spatial and short-term memory. Thereafter, the progressive nature of the disease ensues through complete and irreversible loss of executive function^1^. The most well characterised mechanism associated with neurodegeneration is the amyloid cascade hypothesis of AD. It postulates that the accumulation of the amyloid-β (Aβ) peptides, Aβ_40_ and Aβ_42_ through processing of the amyloid precursor protein (APP) or their decreased removal in the brain result in the formation of aggregated insoluble amyloid plaques^2^. Through a cascade of deleterious effects, the amyloid plaques formed are suggested to break down releasing neurotoxic oligomeric species of Aβ, resulting in synaptic loss and neuronal damage^1,3,4^.

While this theory has been disputed with calls for its reassessment, current consensus supports the hypothesis that the accumulation of the Aβ peptides *in vivo*, contributes to the pathogenesis of both familial and sporadic forms of AD^1,5^. The recapitulation of the mechanisms underlying the onset of AD is most commonly achieved through the use of transgenic mouse models of the disease state^6,7^. These animals typically overexpress mutant human forms of APP and the presenilin genes, triggering the progressive development of many of the pathological hallmarks of AD. Within these transgenic models, APP is overexpressed and is first cleaved by β- followed by γ-secretase, releasing neurotoxic Aβ peptides into the extracellular space.

In addition, Aβ is thought to be transported in an unknown manner from the periphery to the brain as seeds capable of corrupting host Aβ that subsequently deposits as senile plaques^8^. In spite of the extensive use of transgenic animal models, most often they best reflect rare familial forms of AD that may lack the large-scale loss of neurons that follows cognitive decline^9^. While *in vivo* models have provided insight into the pathogenesis of AD, successful therapeutic strategies stemming from their use have thus far, remained elusive. For example, vaccinations using the Aβ_42_ peptide in active immunisation were shown to be effective in both preventing and reversing AD pathogenesis in transgenic mice that overexpressed mutant human APP, in which the normal valine at 717aa was replaced with a phenylalanine residue^6^. However, while these early studies sparked widespread interest it was later found that the use of Aβ_42_ in immunisation regimes triggered excessive inflammation presenting clinically as encephalitis caused by the activation of specific inflammatory T cells^10–14^.

Aβ in immunotherapy utilising active immunisation or passive immunisation of patients using antibodies raised against Aβ have shown therapeutic promise, most notably through reducing the neuropathological hallmarks of AD^6,15^. A recent clinical trial utilising passive immunisation of the monoclonal antibody against aggregated Aβ, aducanumab, demonstrated reductions in both soluble and fibrillar forms of cortical amyloid and a reduction in clinical decline^15^. Phase III clinical trials of the drug are currently underway to uncover new treatment regimes^15^. Overall, immunisation trials continue to highlight the potential of vaccines directed against Aβ in the effective clearance of AD neuropathology.

Recent efforts have focused on the use of three-dimensional (3D) cellular models of AD that were proposed to fully recapitulate Aβ and tau pathology in a single model, thereby overcoming some of the limitations encountered in the use of single transgenic mouse models^16^. Whilst this study was able to demonstrate the presence of amyloid deposits in 3D cell cultures, the study did not provide unequivocal evidence for the deposition of amyloid in a β-pleated sheet conformation, owing to the staining methodologies employed. Paradoxically questions currently remain into how the use of these models will be translated into successful clinical interventions against AD and dementia. This is most probably explained by the lack of knowledge concerning the cellular fate of amyloidogenic species *in vivo* and why normal cellular autophagy processes fail to break down these neurotoxic deposits^17,18^.

Attempts to monitor the extracellular deposition and the subsequent cellular uptake of Aβ, have frequently made use of immunolabelling. Therein, antibodies to Aβ containing a fluorescent tag or immunogold particles of a fixed size, are regularly used to demonstrate intracellular amyloid via fluorescence and transmission electron microscopy (TEM) approaches respectively^19,20^. The specificity of detection in immunolabelling is based on the binding of the primary antibody, of which the paratope is typically directed towards short chain fragments of the full-length peptide^21^. As such, these constructs are able to bind amyloid irrespective of the peptide morphology, rendering them ineffective in tracing the conformational changes of amyloidogenic peptides in complex biological systems.

Commercially available fluorochromes may also be conjugated directly to amyloid either through binding to the N-terminus of the amino acid chain, or as an amine reactive conjugate^21,22^. Use of a fluorochrome ensures an intense fluorescence signal with well-defined excitation and emission properties. However, unlike fluorophores that produce a fluorescence emission only when bound to amyloidogenic peptides, fluorochromes produce fluorescence regardless of whether coordinated or tagged to amyloid. Therefore, it is unclear from these studies as to the conformation of short-chain amyloidogenic species, whether identified intracellularly or deposited extracellularly in simulated animal or cell-based models of vaccination. In order to distinguish between distinct conformational states of amyloid, studies have made use of the isolation and subsequent labelling of amyloid species including monomeric, oligomeric, intermediate protofibril and mature β-pleated sheets of the peptide^20,21^. The inherent ability of amyloid to self-aggregate into a conformation rich in β-sheet structure *in vivo* however, casts doubt on the structural stability of intermediate amyloid species, especially when internalised in complex cellular environments^4,19,20^.

Thioflavin S (ThS) and thioflavin T (ThT) have received frequent use in their ability to identify amyloid deposits by interacting with the cross-β sheet structure of amyloid fibrils^23^. ThT is a benzothiazole dye and ThS is a sulfonated derivative of ThT with similarities to the sulfonated azo dye, Congo red. The inherent lack of selectivity of Congo red binding to amyloid as revealed under fluorescence excitation, however, has impeded its use in the intracellular monitoring of amyloidogenic species^23,24^. Therefore, increasing numbers of studies have used ThT to detect amyloid aggregation within solution, especially when using synthesised or truncated peptides^24–26^.

Interestingly, these classical staining methods for amyloid are often used only to confirm the presence of amyloid, conjugated to its tagged fluorescent label. Herein, we establish direct fluorescent-based labelling methods of Aβ_42_, allowing for the conformation of intracellular amyloidogenic species to be assessed in an *in vitro* T helper 1 (THP-1) cell line. Through the complementary use of TEM, the unequivocal cellular uptake and localisation of amyloid co-adsorbed to clinically relevant adjuvant complexes are demonstrated. These approaches shed light on the cellular fate of Aβ in a simulated cell-based model of vaccination, supporting cellular mechanisms of amyloid presentation in current and future vaccine development.

## Results

### Assessment of the cellular uptake of a model amyloid antigen via direct fluorescent labelling

Thioflavin T (ThT) fluorescence of the amyloidogenic Aβ_42_ peptide was first assessed in R10 cell culture medium, to ensure the formation of mature amyloid fibrils in treatment conditions compatible with subsequent T helper 1 (THP-1) cell culture (see Supplementary methods). Aβ_42_ prepared at 4 μM and incubated over 24 h at 37 °C produced a 64.8% increase in fluorescence intensity measured at 482 nm (13.07 ± 0.34, mean ± SD, *n* = 3), above background of R10 cell culture medium only (4.60 ± 0.85, mean ± SD, *n* = 3). Amyloid fibril formation propagating as β-sheets at the concentrations stated herein was therefore confirmed in R10 media.

**Figure 1:**
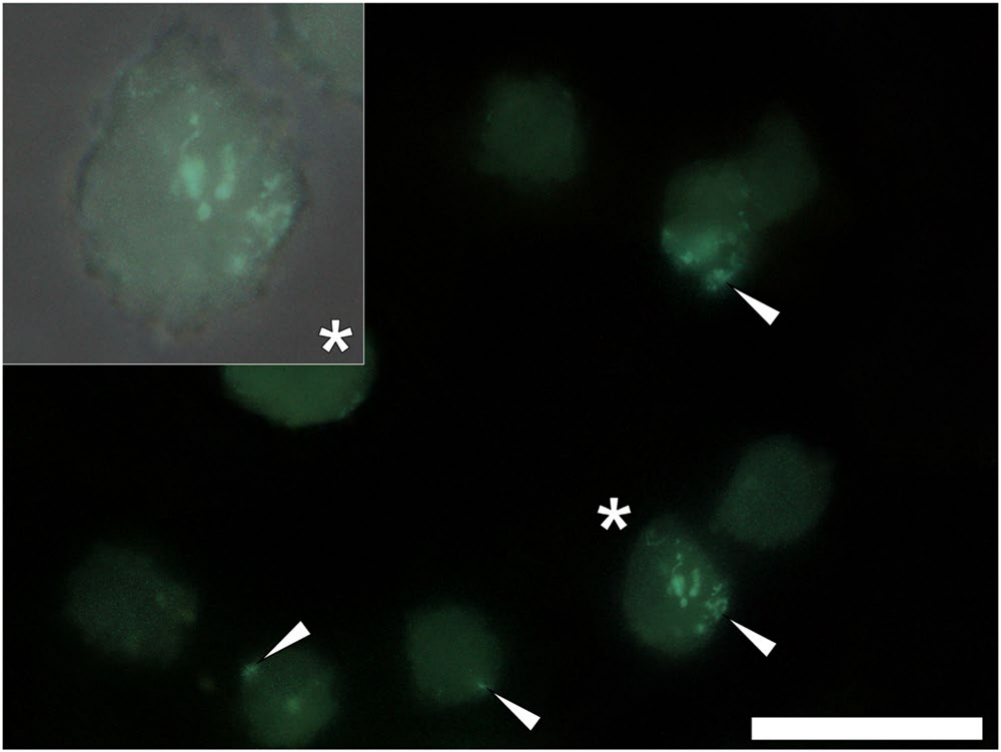
Representative fluorescence microscopy of whole THP-1 cells cultured for 1 h in R10 medium containing 10 μM thioflavin T (ThT) in the presence of 4 μM Aβ_42_. Cells were fixed in 4% *w/v* PFA, washed in 50 mM PIPES buffer, pH 7.4 before mounting with Fluoromount^™^ (Sigma Aldrich) on poly-lysine coated slides. ThT fluorescence (green) is depicted (Olympus long bandpass U-WMBV2 filter cube) with the magnified insert denoted (*) of which the bright-field image has been overlaid. White arrows highlight the presence of ThT-reactive deposits within THP-1 cells. Magnification X 1000, scale bar: 20 μm.

The assessment of the cellular uptake of Aβ_42_ was investigated initially in the absence of added aluminium based adjuvant (ABA) nanomaterials. In order to assess cellular uptake of β-pleated sheets of amyloid formed under these conditions, the use of ThT as a pre-labelling reagent (20 μM) was first assessed in treatments containing 8 μM of the peptide only. Aβ_42_ pre-incubated for 24 h in R10 medium containing ThT and co-cultured (1:1) with THP-1 cells for 1 h (37 °C, 5% CO_2_), revealed the cellular uptake of amyloid in a β-sheet conformation as identified through a green fluorescence emission (482 nm) contained in cell cytosol (Fig. 1). ThT-reactive amyloid was identified following 1 h incubation, as evidenced via punctate green fluorescence in focal deposits at the periphery of THP-1 cells. Cellular uptake of amyloid fibrils was also observed in identical experiments over 1, 3, 6 and 24 h (see Supplementary Fig. 1). THP-1 cells co-cultured under identical conditions in the absence of added amyloid produced a uniform weak green fluorescence emission that was found to increase in intensity upon prolonged incubation over 24 h in the presence of ThT (see Supplementary Fig. 2).

### The complementary use of transmission electron microscopy confirms the cellular internalisation of fibrillar amyloid

**Figure 2:**
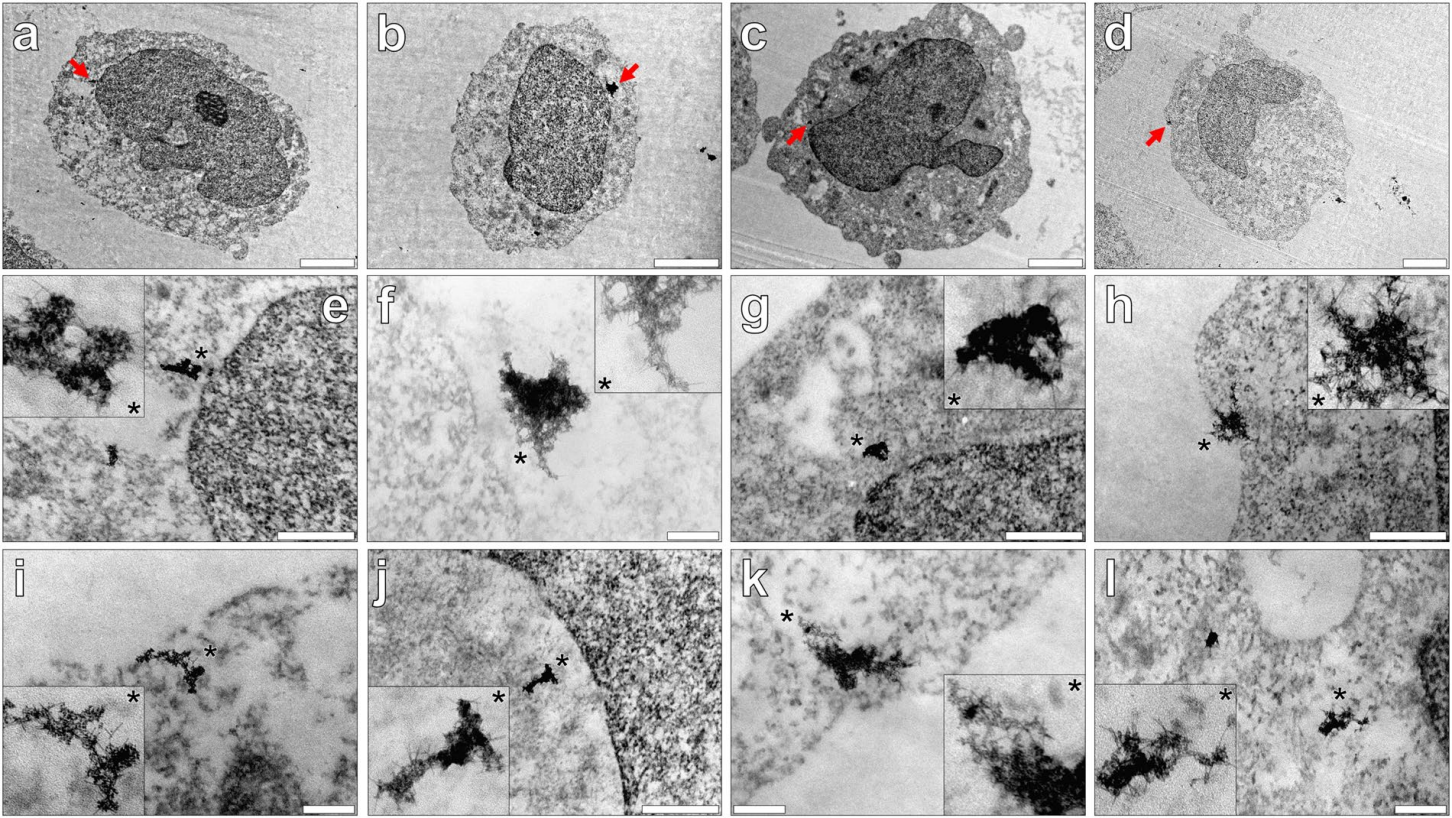
Representative electron micrographs from TEM of Spurr resin-sectioned (100 nm sections) THP-1 cells co-cultured with 4 μM Aβ_42_ in R10 medium containing 10 μM thioflavin T (ThT) for (**a,e** and **i**) 1, (**b,f** and **j**) 3, (**c,g** and **k**) 6 or (**d,h** and **l**) 24 h. Cell resin-sections were stained for 20 min with 2% *w/v* ethanolic uranyl acetate, rinsed with 30% *v/v* ethanol followed by ultrapure water. Grids containing sections were allowed 24 h drying time prior to analysis via TEM. Inserts show close-ups of intracellular negatively stained amyloid deposits and the red arrows highlight their presence within the respective cell images (**e**–**h**). A further example of intracellular negatively stained amyloid fibrils observed at 1, 3, 6 and 24 h, is also depicted (**i**–**l**). Magnification & scale bars: (**a** and **c**). X 10 K, 2 μm, (**b**) X 12 K, 2 μm, (**d**) X 8 K, 2 μm, (**e–h**) X 60 K, 0.5 μm & (**i–l**) X 100 K, 0.2 μm, respectively.

In order to confirm the cellular internalisation of amyloid, THP-1 cells were co-cultured under identical conditions over 1–24 h, of which cell sections (90 nm) were negatively stained with uranyl acetate (2% w/v). Pre- osmium tetroxide and lead citrate staining were omitted to allow for greater clarity in the detection of potential intracellular amyloid. Aβ_42_ aged for 24 h in R10 media and co-cultured with THP-1 cells (at 4 μM) for 1 h, revealed negatively stained deposits of intracellular amyloid fibrils as plaque-like aggregates in cell cytosol (Fig. 2a,e and i). Intracellular clustering of negatively stained fibrils was also identified within the cytoplasm of THP-1 cells following 3 (Fig. 2b,f and j) and 6 h (Fig. 2c,g and k) incubation at 37 °C. Of those fibrillar deposits identified, no aggregates of amyloid were found enclosed in vesicular-like structures in the cytoplasm of THP-1 cells.

At every time point investigated thereafter over 24 h, electron-dense deposits of negatively stained amyloid fibrils were found internalised in THP-1 cells that varied in size dramatically. Mature amyloid fibrils exhibiting classical negative staining were readily identified following 24 h co-culture with cells (Fig. 2d,h and l). Interestingly, negatively stained amyloid fibrils of morphology not dissimilar to intracytoplasmic Aβ_42_ were also found deposited within nuclei of THP-1 cells (see Supplementary Fig. 3). Therefore, TEM of THP-1 cells cultured in the presence of a model Aβ_42_ peptide antigen confirmed the presence and intracellular persistence of amyloid β-sheets.

### Direct fluorescent labelling reveals the cellular internalisation of simulated amyloid adjuvant vaccines

The direct fluorescent labelling of a model Aβ_42_ amyloid antigen (4 μM) and its co-adsorbed ABA (25.0 μg/mL) utilised the fluorophores ThT and lumogallion, respectively. Experiments monitoring cellular uptake of simulated vaccine treatments utilised 20 and 100 μM of the respective fluorophores, throughout. Initially, both fluorescent dyes were added to simulated vaccine treatments at T = 0. All pre-incubated vaccine treatments (24 h) co-cultured 1:1 with THP-1 cells, emitted positive intracellular lumogallion (orange) and ThT fluorescence (green). The broad emission of lumogallion complexed to particulate ABA was, however, found to pass through the longpass fluorescence filter used to collect fluorescence for ThT–reactive amyloid (482 nm), rendering adjuvant and antigen difficult to distinguish from one another (see Supplementary Fig. 4).

In order to isolate the fluorescence of ThT from lumogallion, a single bandpass filter was used (see Supplementary Fig. 5). As such, only emitted light of wavelength 470–500 nm was allowed to pass through to the detector, encompassing the ThT emission maxima of 482 nm when complexed to amyloid. Fluorescence microscopy of aged Aβ_42_ (29 μM), prepared as sections (5 μm) and stained with 1 mM ThT for 24 h, revealed a blue versus a green fluorescence emission under single and long bandpass filters, respectively (see Supplementary Fig. 6). While specificity of ThT to mature amyloid fibrils was observed under both filter types, the fluorescence intensity was found to be weaker under single bandpass emission. However, it was found that fluorescence emission for lumogallion and ThT bound to adjuvant and antigen respectively, could be successfully isolated thereby allowing for their intracellular identification within THP-1 cells (see Supplementary Fig. 5). Therefore, single bandpass emission filters were used throughout for the detection of intracellular fluorescence of antigen and adjuvant formulations in simulated vaccine treatments.

Optimisation of pre-labelling simulated vaccine formulations focused upon obtaining distinguishable fluorescence of amyloid co-adsorbed to its ABA, over the shortest possible time period. Simulated vaccine treatments containing 8 μM Aβ_42_ in the presence or absence of 25.0 μg/mL of an ABA (Alhydrogel®, Adju-Phos® or Imject™ Alum) were first incubated for 24 h, prior to their addition to THP-1 cells. Pre-incubated treatments were subsequently plated 1:1 with cells for 24 h, of which the fluorophores ThT and lumogallion were simultaneously added only 3 h prior to fixation, in order to minimise their influence on the cellular uptake of Aβ_42_ and co-adsorbed ABA.

**Figure 3:**
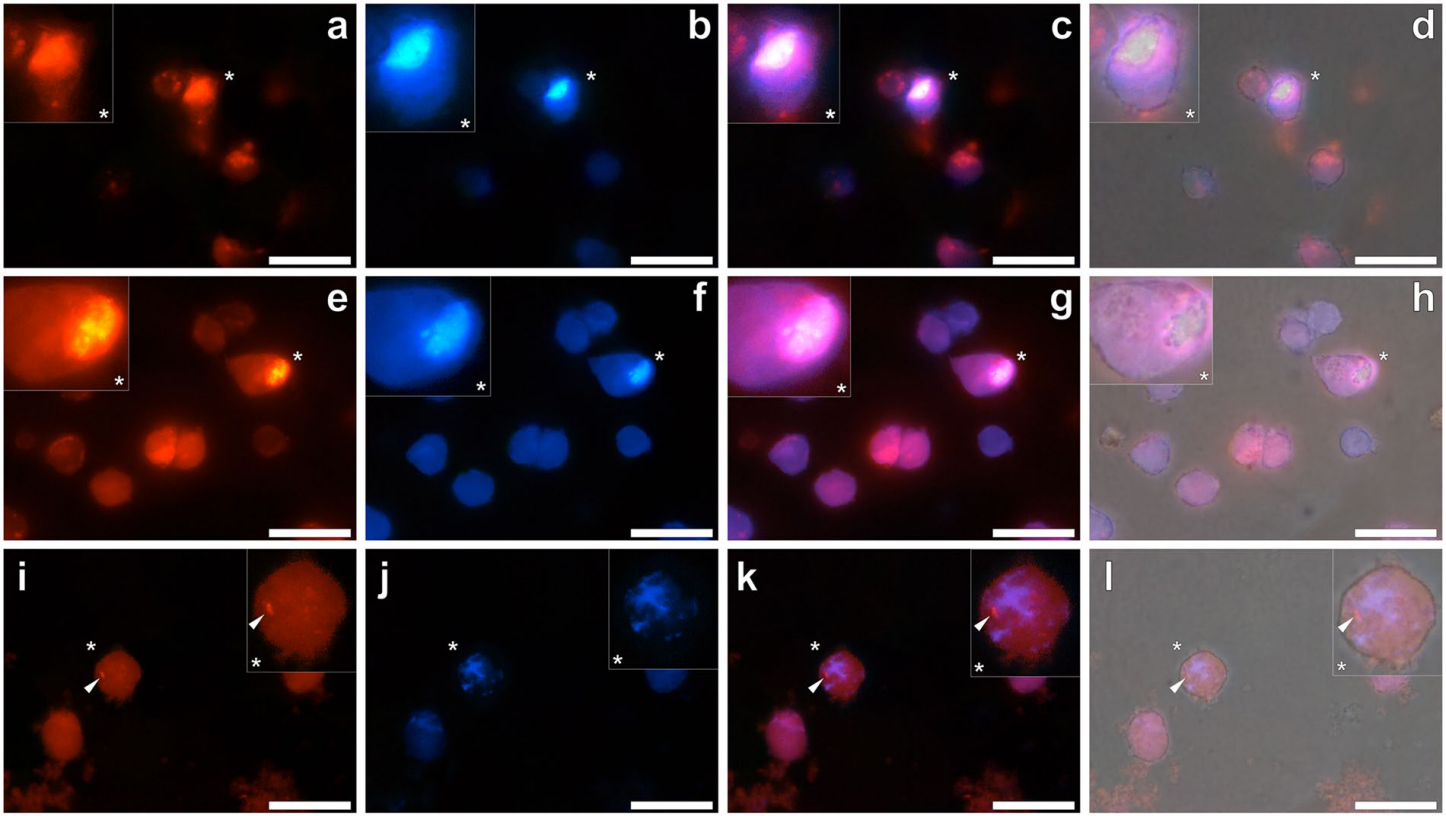
Representative fluorescence microscopy of whole THP-1 cells cultured for 24 h in R10 medium in the presence of a simulated vaccine formulation. Vaccine treatments were prepared via the addition of 8 μM Aβ_42_ and 25 or 12.5 μg/mL ABA to complete R10 medium. Following 21 h incubation at 37 °C, 20 μM ThT and 100 μM lumogallion was added to the respective treatments and incubated for a further 3 h, prior to their 1:1 addition to THP-1 cells. Cells co-cultured for 24 h with simulated vaccine treatments contained 4 μM Aβ_42_ and (**a–d**) 12.5 μg/mL Alhydrogel^®^ (Brenntag), (**e–h**) 12.5 μg/mL Imject Alum™ (Pierce, Thermo Scientific) or (**i–l**) 6.25 μg/mL Adju-Phos^®^ (Brenntag). Cells were fixed in 4% *w/v* PFA, washed in 50 mM PIPES buffer, pH 7.4 before mounting with Fluoromount^™^ (Sigma Aldrich) on poly-lysine coated slides. Lumogallion (orange) (**a**,**e** and **i**) and ThT (blue) fluorescence (**b**,**f** and **j**) is depicted (Olympus single bandpass U-WMBV2 filter cube) with magnified inserts denoted (*). Merged lumogallion and ThT channels (**c**,**g** and **k**), combined with the light overlay (**d**,**h** and **l**) are also depicted. Arrows highlight discreet adjuvant particles. Magnification X 1000, scale bars: 20 μm.

THP-1 cells co-cultured in the presence of pre-labelled Aβ_42_ only produced a blue fluorescence emission within the cytoplasm of THP-1 cells. In the absence of added ThT, THP-1 cells co-cultured under identical conditions produced a uniform blue fluorescence emission of lower relative intensity (see Supplementary Fig. 7). Simultaneous pre-labelling of Aβ_42_ in the presence of Alhydrogel® revealed an intracellular orange fluorescence emission under the lumogallion fluorescence channel, indicative of particulate loading in cell cytosol (Fig. 3a). Upon illumination of the same population of whole cells under the ThT fluorescence channel, an intense intracellular blue fluorescence was observed (Fig. 3b). Merging of both lumogallion and ThT fluorescence filter channels for THP-1 cells co-cultured with Aβ_42_ adjuvanted with Alhydrogel® demonstrated the co-localisation of amyloid and adjuvant material, of which merging of both signals resulted in a pink-purple fluorescence emission (Fig. 3c). Furthermore, overlaying of the bright field image confirmed that ThT and lumogallion reactive material was confined to THP-1 cells (Fig. 3d).

For those cells co-cultured with vaccine treatments containing Aβ_42_ in the presence of Imject™ Alum, lumogallion-reactive particulates were identified in cell cytosol via an intensive orange fluorescence emission (Fig. 3e). ThT-reactive material was additionally identified intracellularly via the observation of a blue fluorescence emission confined to THP-1 cells (Fig. 3f). As with cells treated with amyloid and adjuvanted with Alhydrogel^®^, both lumogallion and ThT fluorescence was found co-localised in the cytoplasmic compartment of cells (Fig. 3g). Merging of bright field and fluorescence channels indicated that ThT and lumogallion positive fluorescence was confined to THP-1 cells (Fig. 3h).

Simulated Aβ_42_ vaccine treatments adjuvanted with 25.0 μg/mL Adju-Phos^®^ and co-cultured with THP-1 cells, produced an intensive and uniform intracellular orange fluorescence emission, under the lumogallion filter channel. Due to the inability to distinguish lumogallion reactive material in whole cells, the concentration of Adju-Phos^®^ was halved in cell culture conditions. Subsequent analyses of co-cultured THP-1 cells highlighted the presence of a punctate intracellular orange lumogallion-reactive fluorescence emission (Fig. 3i). A blue fluorescence emission was also observed in identical cells under the ThT fluorescence channel and in close proximity to those structures producing lumogallion fluorescence (Fig. 3j). Interestingly ThT fluorescence was identified infrequently and of a lower intensity in comparison to those cells co-cultured in the presence of Alhydrogel^®^ and Imject™ Alum, respectively. Merging of fluorescence channels revealed the close proximity of ThT and lumogallion fluorescence within the cytoplasm of THP-1 cells (Fig. 3k), of which overlaying of the bright field image confirmed their co-deposition in cells (Fig. 3l).

### Confirmation of the cellular internalisation of amyloid and aluminium based adjuvant materials via transmission electron microscopy

The complementary method of TEM was performed in addition to fluorescence microscopy, in order to support the observations of intracellular Aβ_42_ co-adsorbed to its respective ABA. THP-1 cells were co-cultured with simulated amyloid vaccines under near-identical conditions and pre-labelling regimes, of which the ABA concentration was maintained at 50.0 μg/mL across treatment conditions. This concentration of ABA has proven optimal in our previously published studies of adjuvant uptake in a THP-1 cell line^27,28^ and hence, was selected herein.

**Figure 4:**
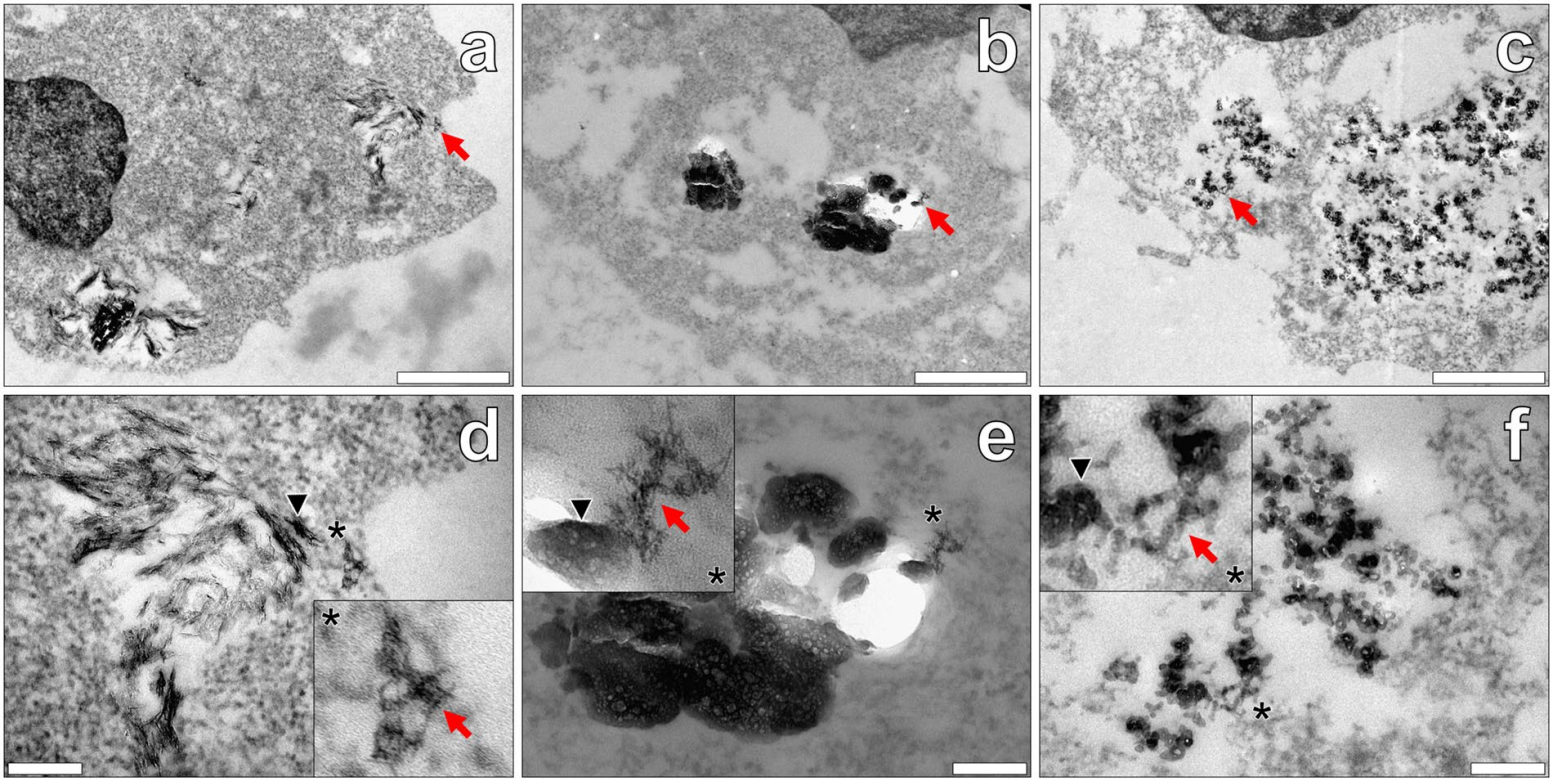
Representative electron micrographs from TEM of Spurr resin-sectioned (100 nm sections) THP-1 cells co-cultured with simulated vaccine formulations. Cells were co-cultured for 24 h in R10 medium with 4 μM Aβ_42_ in the presence of 50 μg/mL Alhydrogel® (Brenntag Biosector, Denmark) (**a** and **d**), Imject™ Alum (Pierce, Thermo Scientific) (**b** and **e**) or Adju-Phos® (Brenntag, Denmark) (**c** and **f**). ThT and lumogallion at 10 and 50 μM respectively, were added at 21 h, as with fluorescence analyses of THP-1 ABA co-cultures. Cell resin-sections were stained for 20 min with 2% w/v ethanolic uranyl acetate, rinsed with 30% v/v ethanol followed by ultrapure water. Grids containing sections were allowed 24 h drying time prior to analysis via TEM. Inserts show close-ups of intracellular negatively stained amyloid (red arrows) co-deposited with ABA (black arrows) within the respective cell images. Magnification & scale bars: (**a–c**) X 30 K, 1 μm, (**d–f**) X 100 K, 0.2 μm, respectively.

THP-1 cells co-cultured with simulated amyloid vaccines adjuvanted with Alhydrogel^®^ revealed semi-crystalline and electron dense needles found solely within vesicular-like compartments within cell cytoplasm (Fig. 4a). Higher magnifications revealed negatively stained amyloid fibrils found deposited within cell cytosol. Interestingly, fibrillar deposits of apparent amyloid were identified both in the absence and presence of co-deposited needles of the adjuvant (Fig. 4d). Although rarely observed, negatively stained amyloid fibrils were found localised in cell nuclei, as with those experiments performed in the presence of the peptide only (see Supplementary Fig. 3). THP-1 cells co-cultured in simulated vaccine treatments containing Imject™ Alum, demonstrated the intracellular presence of large amorphous plate-like particulates of the adjuvant, spanning several microns in width. Those electron-dense aggregates identified were contained in vesicular-like compartments and found solely within the cytoplasm of THP-1 cells (Fig. 4b). Negatively stained amyloid fibrils were observed only in the cell cytoplasm of THP-1 cells loaded with particulate adjuvant. The direct co-localisation of fibrillar amyloid and amorphous plates of particulate adjuvant material were clearly identified via negative staining with uranyl acetate (Fig. 4e).

Finally, analyses of THP-1 cells co-cultured with Adju-Phos^®^ in the presence of Aβ_42_ revealed the clear presence of positively stained amorphous aggregates in endosomal-like compartments (Fig. 4c). While particulate ABA was clearly contained intracellularly, classical negative staining of fibrillar amyloid was elusive. However, fibril-like projections formed between adjacent particulates were identified contained within the cytoplasm of THP-1 cells.

## Discussion

We have investigated the fate of amyloid as a model antigen in a cellular model of vaccination. Through the use of direct fluorescent labelling, the intracytoplasmic accumulation of Aβ_42_ propagating as β-sheets was found in a monocytic THP-1 cell line. The use of ThT as a conformation-specific fluorophore for β-sheets of Aβ_42_ revealed that cytosolic loading of the amyloidogenic peptide occurred within 1 h and persisted intracellularly over 24 h, as revealed by a green intracellular fluorescence emission^24^.

A recent study monitoring the cellular uptake of Aβ_42_ identified the clathrin-independent endocytic route of macropinocytosis that allowed for the entry of the amyloidogenic peptide into a neuroblastoma SH-SY5Y cell line^21^. As a known pathway for the cellular uptake of protein antigens, solute molecules and nutrients, internalisation via this route is rapid as with phagocytosis occurring within minutes upon exposure to cultured cells^29,30^. The subsequent fusing of macropinocytotic vesicles with late lysosomes results in an acidic protease-rich environment, capable of degrading their vesicular cargo^30^.

As a high resolution and complementary technique to fluorescence microscopy, TEM of sectioned THP-1 cells successfully identified intracytoplasmic negatively stained amyloid fibrils. Interestingly, while the latter has been shown intraneuronally *in vivo*, their enclosure in endosomal-like vesicles was not observed under the dosing regimen utilised herein^20,31^. Therefore, our results indicate the absence of mature autophagolysosomes, typically demonstrated through the presence of membrane-enclosed vesicles via TEM^27,31^.

In support of our new findings, recent studies have shown that while the cellular uptake of amyloid fibrils mechanistically initiates autophagy, the resultant degradation of amyloid is hindered through rupturing of lysosomal compartments^32,33^. Han *et al*., (2017) employed scanning transmission electron microscopy (STEM) that revealed the intracellular tomography of fibrillar assemblies of Aβ_40_ in THP-1 cells. Therein, pre-incubation of the peptide (20 μM) with non-differentiated THP-1 cells for 48 h, resulted in bundles of amyloid fibrils penetrating tubular invaginations of the phospholipid bilayer^32^. Such was further supported through increases in propidium iodide fluorescence upon binding to extracellular DNA, released upon perforation of the plasma membrane. Lysosomal leakiness of THP-1 cells was subsequently confirmed upon co-culture with Aβ_40_, indicated via the reduction of acridine orange upon release from acidified vesicles to the cell cytoplasm^32^.

More recently, fibrillar forms of the Parkinson’s disease (PD) related amyloidogenic protein, α-synuclein, were found to induce rupture of intracellular vesicles, following endocytosis^33^. Interestingly, amyloid fibrils produced from both wild-type and mutant familial related variants of the protein were observed to rupture lysosomal vesicles whether co-cultured with neuroblastoma SH-SY5Y or human dopaminergic neuronal cell lines. Vesicle rupture of the former was also noted when co-cultured with the human AD-related microtubule-associated protein, tau^33^. Therefore the propensity of all amyloidogenic proteins to undergo structural conversion from their soluble native α-helical form to a conformation rich in β-sheet structure, pre-disposes their fibrillar structure to rupture intracellular lysosomal vesicles upon uptake^32,33^.

Aβ_42_ of increasing size was additionally identified in a β-pleated sheet conformation within cell nuclei over prolonged incubation periods. The presence of nuclear inclusions containing amyloid in neurodegenerative disorders has revealed co-localisation of the eukaryotic protein, ubiquitin^17,34^. As an important initiator of the proteasomal machinery in intracellular environments, covalent conjugation of the protein to misfolded proteins would normally lead to their degradation, *in vivo*^17^. In AD, ubiquitin is known to persist in such nuclear protein inclusion bodies of which the accumulation of long-chain ubiquitin molecules only has been shown to form amyloid-like fibrils, potentially exacerbating the condition^17^.

The persistence of intracytoplasmic or intranuclear amyloid may also be explained through disruptions in protein homoeostasis^31^. The mammalian target of rapamycin complex 1 (mTORC1) is a key regulator of autophagy of which its inactivation allows for nutrient metabolism and the removal of non-functional and misfolded proteins^35^. In AD however, downstream mTORC1 signalling through phosphoinositide-3-kinase (PI3K), leads to the accumulation of autophagic vacuoles^36^. Therefore, our observation of cellular accumulation of the peptide antigen only depositing as mature amyloid fibrils, possibly owed to the inability of THP-1 cells to degrade these aberrant deposits.

The direct fluorescent labelling of a model Aβ_42_ peptide antigen formulated in the presence of clinically relevant and experimental ABA preparations revealed their co-localisation in the cytosol of THP-1 cells. Lumogallion is an established and sensitive fluorescent molecular probe for the identification of aluminium in both cells and tissues and was utilised herein to monitor the fate of ABA, co-adsorbed to its target peptide antigen^28,37,38^. The complementary technique of TEM supported the observations from fluorescence microscopy, revealing the co-uptake of ABA and aggregates of β-pleated sheet amyloid antigen materials, in sectioned THP-1 cells.

Furthermore, TEM confirmed the presence of needle-like crystals of Alhydrogel^®^, plate-like aggregates of Adju-Phos^®^ and amorphous deposits of Imject Alum™, all found enclosed within endosomal intracellular compartments. Therefore, the cellular uptake of both the clinically approved and experimental adjuvant formulations was in agreement with our previous work, even when coadministered with a model Aβ_42_ peptide antigen^27^.

Negatively stained amyloid fibrils of the model antigen were clearly distinguishable in cell cytosol when co-administered with Alhydrogel^®^ and Imject Alum™. Interestingly, mature fibrillar aggregates co-deposited with Alhydrogel^®^ or directly attached to Imject Alum^®^ were identified on the periphery of endocytosed particulate adjuvant materials. However, intracellular aggregates of mature Aβ_42_ were elusive in vaccine formulations adjuvanted with Adju-Phos^®^ in which the potential co-localisation of amyloid fibrils and ABA could only be detected at higher magnifications.

Intracytoplasmic vesicles noted for Aβ_42_ formulated in the presence of ABA, indicated that endocytosis was the most likely route of entry through autophagic processing to lysosomes^27,37,39^. As with THP-1 cells co-cultured with Aβ_42_ only however, mature amyloid fibrils whether co-localised or co-adsorbed to their target ABA, were not observed to enter endosomal-like vesicles. Therefore, cellular uptake of Aβ_42_ as a model peptide antigen and ABA are suggested to be internalised via the differing endocytic pathways of macropinocytosis and autophagy, respectively^21,29,32,33^. To our knowledge, this is the first report of the apparent lack of lysosomal vesicular enclosure of the pathogenic Aβ_42_ species in a simulated cell-based vaccination model of AD.

The thermodynamic saturation constant of Aβ_42_ predicts that spontaneous amyloid fibril formation occurs at concentrations at or exceeding 2 μM^40^. As such, catalysis of Aβ through metal interactions, glycoprotein binding and seeding have been implicated in the induction of auto polymerisation of nanomolar concentrations of the peptide, typically found *in vivo*^40–42^.

Furthermore, templating of soluble Aβ_40_ *in vitro* has been found to undergo polymerisation to a mature fibrillar form, upon the addition of brain extracts containing amyloid fibrils from transgenic AD mice^42^.

More recently, corrupting of host Aβ *in vivo* in the brains of transgenic murine models of AD has been demonstrated through intracerebral inoculation with brain extracts containing fibrillar amyloid^8,43^. Interestingly, Aβ injected intraperitoneally was found to elicit a Trojan horse like mechanism, delivering Aβ as seeds to the brain of transgenic murine models of AD^8^. Regardless of the mechanism by which self-aggregation occurs, repeated nucleation of the soluble alpha-helical form of the peptide through nucleation-driven seeding results in the self-propagation of amyloid to a conformation rich in β-sheet structure^1,3,18^.

Therefore, our results support the uptake and potential transport of amyloid in a β-pleated sheet conformation that persists intracellularly, speculatively through lysosomal degradation^32,33^ accumulating and propagating as plaque-like fibrillar deposits. Circulating blood-borne monocytes are known to enter the central nervous system (CNS) in response to neuroinflammation and are thought to promote tissue repair and support the production of neurotrophic growth factors^44^. The intracellular persistence of mature amyloid fibrils through evasion of autophagy or proteasomal-degradation mechanisms may therefore also allow for the silent entry of fibrillar amyloid across the blood-brain barrier (BBB).

Monocytes are one of the first phagocytic antigen presenting cell (APC) types to be recruited to the site of injection, following vaccination^45^. As such, mechanisms governing the immunoreactivity and cellular fate of amyloid may prove key in the development of vaccination regimes targeting these aberrant deposits. In summary, we herein demonstrate the ability for β-pleated sheet-rich amyloid fibrils to persist intracellularly, of which their translocation away from the injection site could be enhanced in the presence of clinically relevant ABA. Alhydrogel^®^ has previously been demonstrated as a more effective adjuvant for translocation away from the injection site, through its heightened cellular loading in THP-1 cells versus Adju-Phos^®^. The apparent lack of mature amyloid fibrils found for cells co-cultured with the latter would further implicate such *in vivo*^27^.

In conclusion, we have demonstrated the use of direct fluorescent labelling in the cellular monitoring of amyloid when administered in simulated vaccine formulations. The ability to detect both an intracellular amyloid antigen and its co-adsorbed ABA following their internalisation, provide proof of concept for the transport of both fluorophores across the cell membrane of THP-1 cells^37^. Furthermore, the conformation-specific fluorophore, ThT, clearly demonstrated the cellular persistence of misfolded amyloid deposits in both cytosolic and nuclear inclusions. Overall, direct fluorophore-labelling and high-resolution TEM approaches omitting usual lead citrate staining may provide invaluable tools in the assessment of existing and future vaccinations directed against amyloid and other relevant neuropathological hallmarks of wider dementia.

## Methods

### Preparation of Aβ_42_ stock solutions

All chemicals were purchased from Sigma Aldrich, UK unless otherwise stated. The amyloidogenic peptide, Aβ_42_ was purchased from Bachem as the lyophilised salt and was reconstituted in 0.01 M NaOH to prepare 100 μM stocks. Under these highly alkaline conditions (*ca* pH 12), the fully dissolved peptide only exists in a monomeric form^25,46^. Subsequent stock solutions were aliquoted and stored frozen (−20 °C) and the peptide was thawed fully, immediately prior to use. Peptide concentrations in the final stock solutions were determined prior to their dilution in complete R10 medium. Briefly, thawed stocks were centrifuged at high speed (5 min, 15000 *g*) and their protein content determined using absorbance at 280 nm using a NanoDrop 1000 spectrophotometer (Thermo Scientific, UK). The final measured peptide concentration was adjusted based on percentage purity stated by the manufacturer and was used to prepare subsequent dilutions of Aβ_42_.

### Preparation of simulated vaccine formulations

Herein, the clinically relevant aluminium based adjuvants (ABA), Alhydrogel^®^ (aluminium oxyhydroxide) and Adju-Phos^®^ (aluminium hydroxyphosphate) were utilised (both from Brenntag Biosector, Denmark) in formulating simulated vaccines. Dilutions of the ABA were made in a simulated vaccine diluent of 0.9% *w/v* NaCl (autoclaved and sterile filtered through a 0.22 μm membrane) at 1.0 mg/mL, of which further dilutions were made into the respective treatments^27^. Aβ_42_ was prepared in the absence or presence of a given ABA in R10 medium. Treatments were prepared aseptically in a cell culture hood and consisted of antigen only: 8 μM Aβ_42_, adjuvant only: 25.0 or 12.5 μg/mL ABA, or antigen and adjuvant. All treatments were incubated at 37 °C in a dedicated cell incubator for 24 h in order to promote amyloid fibril formation of Aβ_42_, prior to their addition to cells. Amyloid fibril formation was confirmed in R10 medium as described in the Supplemental methods.

### Direct fluorescent pre-labelling of amyloid vaccine co-cultures

THP-1 cells were cultured to a density of 1.0 × 10^6^ cells/mL of which 100 μL per well (*ca* 50,000 cells) were transferred into 96 well plates (TC treated, VWR, Corning Costar^®^) (see Supplemental methods). Cell viability was confirmed by use of the Trypan blue (Life Technologies, UK) exclusion test. Native THP-1 cells containing neither additional ABA nor antigen were diluted 1:1 with R10 medium. Pre-incubated treatments were diluted 1:1 with cells to a final volume of 200 μL, thereby providing simulated vaccines containing 4 μM Aβ_42_ antigen in the absence or presence of 12.5 or 6.25 μg/mL ABA only, in the final co-culture conditions.

Initial experiments, monitoring the cellular uptake of Aβ_42_ only (longpass emission), were formulated in R10 medium containing 20 μM ThT at T = 0. Subsequent treatments prepared in this manner were added to THP-1 cells following 24 h incubation. Experiments utilising treatments containing both Aβ_42_ and an additional ABA were plated with THP-1 cells and incubated for 21 h at 37 °C (5% CO_2_). Dyes for pre-labelling utilised thioflavin T (ThT) at 100 μM and lumogallion at 1 mM, both prepared in ultrapure water and filtered through 0.22 μm syringe filters. ThT and lumogallion were added to cells co-cultured with simulated vaccine treatments at a final concentration of 10 and 50 μM respectively and treated cells were incubated for a further 3 h, prior to fixation.

### Fluorescence microscopy

Whole non-sectioned THP-1 cells were prepared on poly-lysine coated slides, as described in the Supplemental methods. Fluorescence micrographs were obtained by use of an Olympus BX50 microscope equipped with a BX-FLA reflected light fluorescence attachment (mercury source) and a vertical illuminator. Micrographs obtained at X 1000 magnification utilised an X 100 Plan-Fluorite oil immersion objective (Olympus, UK) using low auto-fluorescence immersion oil (Olympus immersion oil type-F).

Fluorescence filter cubes utilised a U-MNIB3 cube (excitation: 470–495 nm, dichromatic mirror: 505 nm, Olympus, UK) with the emission filter swapped for a single bandpass ET590/33 m filter (emission: 570–610 nm, Chroma^®^, Vermont, US) for lumogallion imaging. ThT-reactive amyloid was imaged using a U-MWBV2 cube in long bandpass mode utilising the original mirror unit (excitation: 400–440 nm, dichromatic mirror: 455 nm, longpass emission filter: 475 nm) and in single bandpass mode utilising an ET480/30 m filter (emission: 470–500 nm, Chroma^®^, Vermont, US). Light transmission values were fixed (1000 ms) across respective treatment conditions and images were acquired using the Cell^D^ (Olympus, Soft Imaging Solutions, GmbH) software package.

### Transmission electron microscopy

Spurr-resin embedded agar-cell blocks were sectioned using cut glass knives at 90 nm, by use of an automated REICHERT-JUNG Ultracut E ultramicrotome. Any sections containing ABA materials were sectioned at 100 nm by use of a Leica ultracut UCT ultramicrotome, equipped with a 45° Diatome diamond knife (30-US, Electron Microscopy Sciences). Sections were mounted on G2002 200 mesh thin bar copper grids (Athene, Agar Scientific, UK), pre-treated with a COAT-QUICK “G” grid coating pen (Daido Sangyo Co. Ltd. Japan) for adherence. Grids were allowed 24 h drying time prior to analysis. Cell sections for TEM were viewed on a JEOL 1230 transmission electron microscope operated at 100 kV (spot size 1). A 10 μA activated field emission was used to increase the standing current to 67–68 μA during image acquisition. Electron micrographs were captured using a Megaview III digital camera from Soft Imaging Systems (SiS), using the iTEM universal TEM imaging platform software.

### Data availability

All data generated or analysed during this study are included in this published article and its Supplementary Information files.

## Electronic supplementary material

Supplementary information
