# Supplementary material for: Intracellular tracing of amyloid vaccines through direct fluorescent labelling
Source: Sci Rep. 2018 Feb 5;8:2437. doi: 10.1038/s41598-018-20845-9 (PMC5799327; doi:10.1038/s41598-018-20845-9)
Supplement: Supplementary file 1 — Supplementary information [file 41598_2018_20845_MOESM1_ESM.pdf]

Supplementary information

Title:

Intracellular tracing of amyloid vaccines through direct fluorescent labelling.

Authors: Matthew Mold, Manpreet Kumar, Ambreen Mirza, Emma Shardlow & Christopher Exley*****.

Postal address:

The Birchall Centre, Lennard-Jones Laboratories, Keele University, Keele, Staffordshire, ST5 5BG, UK.

Corresponding author:

Prof. Christopher Exley: c.exley@keele.ac.uk

Supplementary figures

Thioflavin T (ThT) staining of T helper 1 (THP-1) cells at 1, 3, 6 and 24 h highlighting the presence of intracellular amyloid fibrils identified via a green fluorescence emission.

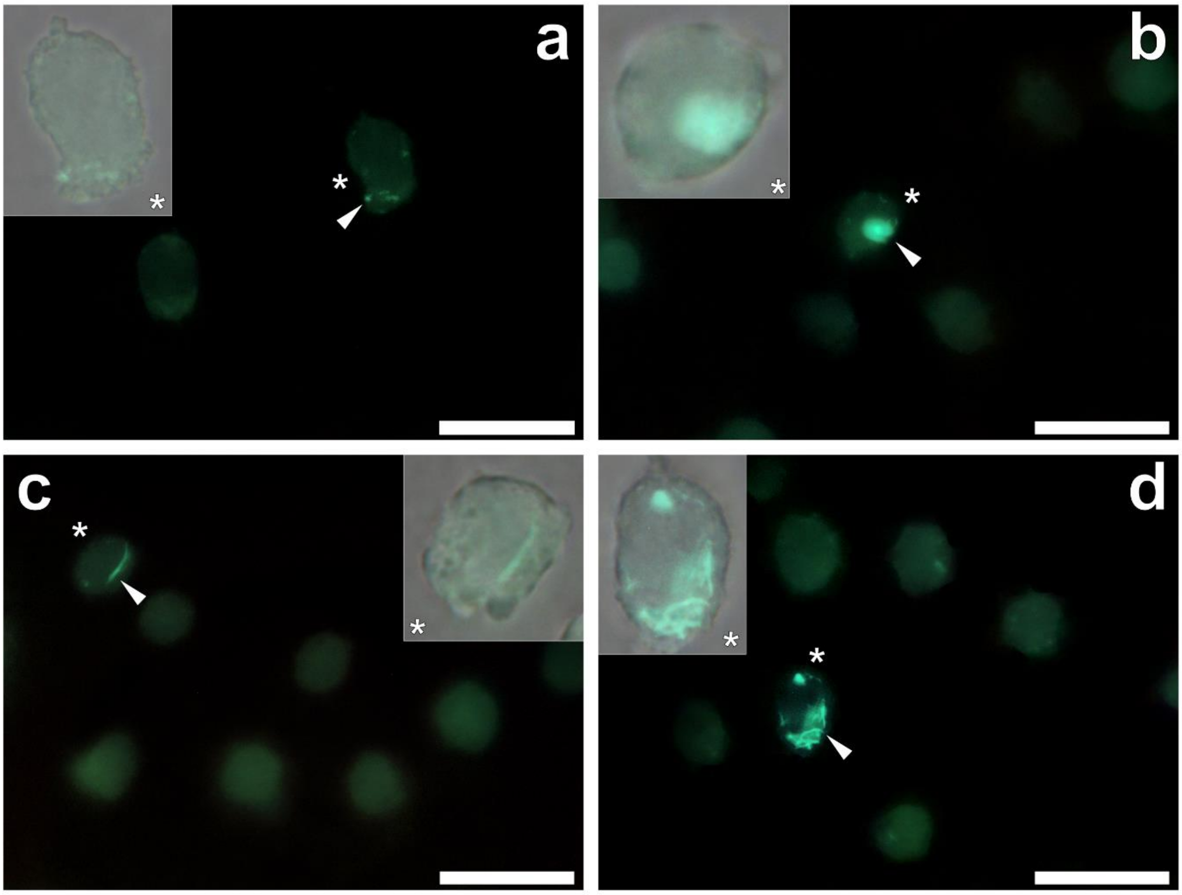

Supplementary Figure 1 Representative fluorescence microscopy of whole THP-1 cells cultured for (**a**) 1, (**b**) 3, (**c**) 6 or (**d**) 24 h in R10 medium containing 10 μM thioflavin T (ThT) in the presence of 4 μM Aβ_{42}. Cells were fixed in 4% *w/v* PFA, washed in 50mM PIPES buffer, pH 7.4 before mounting with Fluoromount™ (Sigma Aldrich) on poly-lysine coated slides. ThT fluorescence (green) is depicted (Olympus long bandpass U-WMBV2 filter cube) with magnified inserts denoted (*) of which the bright-field image has been overlaid. Arrows highlight the presence of ThT-reactive deposits within THP-1 cells. Magnification X 1000, scale bars: 20 μm.

ThT staining of native THP-1 cells in the absence of added amyloid reveal a weak green fluorescence emission.

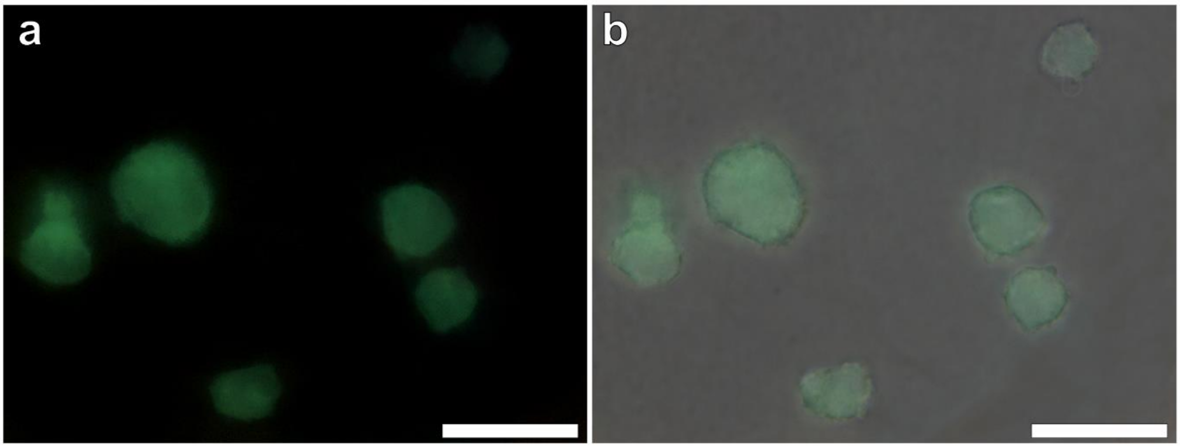

Supplementary Figure 2 Fluorescence micrographs of native THP-1 cells cultured for 24 h in R10 medium containing *ca* 10 µM ThT. Fixed intact cells were mounted on poly-lysine coated slides, mounted with Fluoromount™ (Sigma Aldrich) and viewed under a U-MWBV2 (Olympus, UK) fluorescence filter cube (excitation: 400 – 440 nm, dichromatic mirror: 455 nm, longpass emission: 475 nm). A uniform green fluorescence emission was observed (**a**) contained to THP-1 cells only as revealed by overlaying of the bright-field image (**b**). Magnification X 1000, scale bars: 20 µm.

Intranuclear amyloid in THP-1 cells is clearly identified through negative staining utilising transmission electron microscopy.

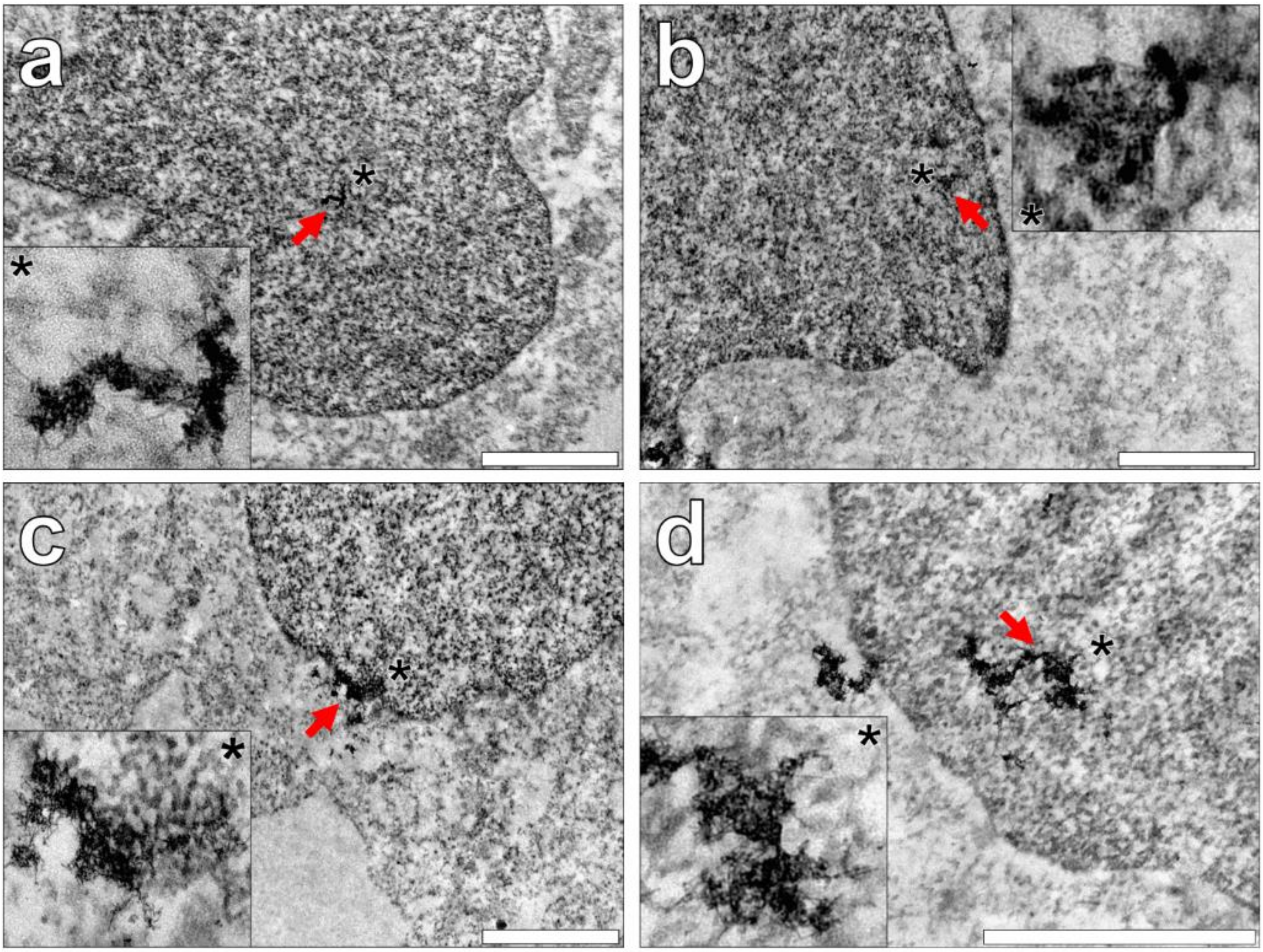

Supplementary Figure 3 Representative electron micrographs from TEM of Spurr resin-sectioned (100 nm sections) THP-1 cells. Cells were co-cultured with *ca* 4 µM $A\beta_{42}$ in R10 medium containing 10 µM thioflavin T (ThT) for (**a**) 1, (**b**) 3, (**c**) 6 or (**d**) 24 h. Cell resin-sections were stained for 20 min with 2% *w/v* ethanolic uranyl acetate, rinsed with 30% *v/v* ethanol followed by ultrapure water. Grids containing cell sections were allowed 24 h drying time prior to analysis via TEM. Inserts show close-ups of intranuclear negatively stained amyloid fibrils and the red arrows highlight their presence within the respective cell images. Magnification & scale bars: **a – c.** X 30 K, **d.** X 60 K, scale bars: 1 µm, respectively.

Modifications made to an existing Olympus U-MWBV2 fluorescence filter cube, allow for the isolation of lumogallion (orange) and ThT (blue) fluorescence, for amyloid and ABA, respectively.

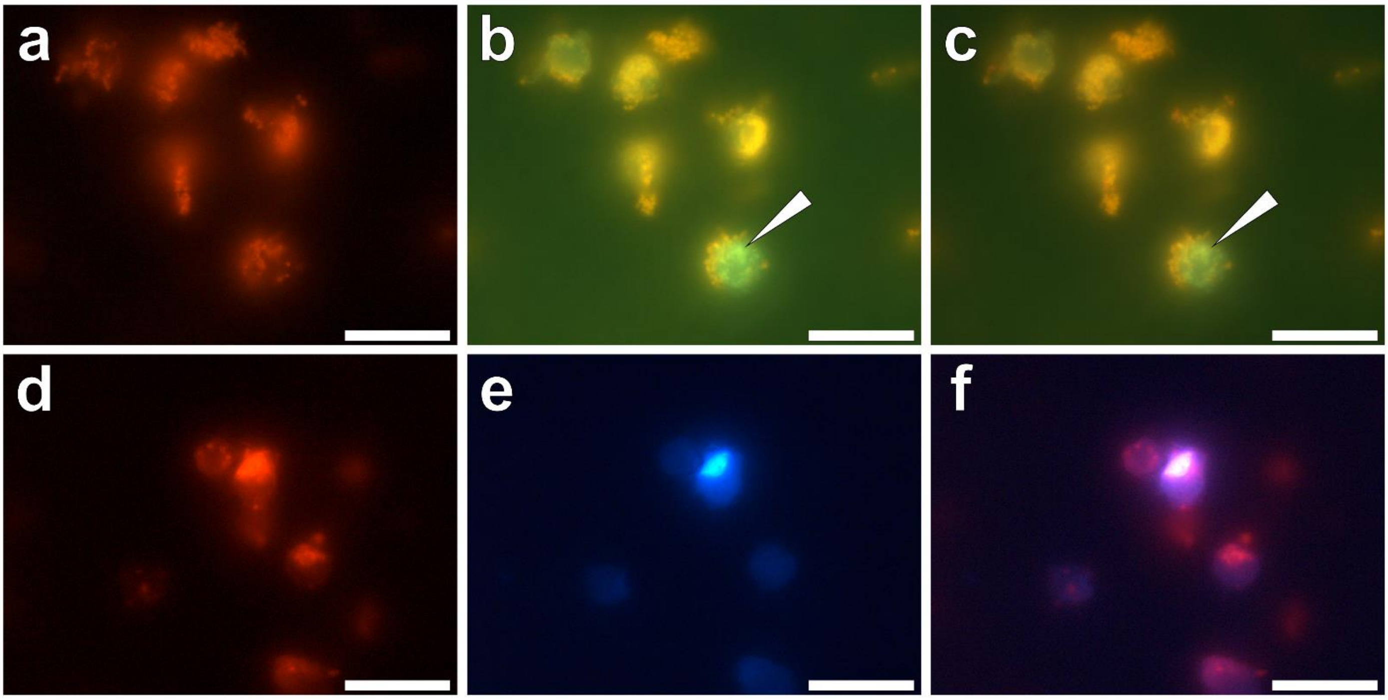

Supplementary Figure 4 Fluorescence microscopy depicting cellular uptake of a simulated vaccine formulation containing an aluminium oxyhydroxide Alhydrogel® (Brenntag, Denmark) adjuvant and an Aβ_{42} peptide antigen. THP-1 cells were cultured for 24 h in R10 medium containing *ca* 4 µM Aβ_{42} and 25 (**a** – **c**) or 12.5 µg / mL Alhydrogel® (**d** – **f**). ThT at *ca* 10 µM and lumogallion at *ca* 50 µM were prepared in ultrapure water and added to cells at T = 0 (**a** – **c**) or T = 21 h (**d** – **f**) for a total incubation period of 24 h. Lumogallion fluorescence (orange) (**a** & **d**) was acquired by use of a U-MNIB3 (excitation: 470 – 495 nm, dichromatic mirror: 505 nm) fluorescence filter cube (Olympus, UK), fitted with an ET590/33m single bandpass emission (570 – 610 nm) filter (Chroma®, Vermont, US). ThT fluorescence was acquired using an Olympus U-MWBV2 filter cube in longpass (green) (**b** & **c**) (excitation: 400 – 440 nm, dichromatic mirror: 455 nm, longpass emission: 475 nm) or single bandpass (blue) mode (**e** & **f**) via use of a Chroma® ET480/30m single bandpass emission (470 – 500 nm) filter. Lumogallion and ThT overlays are also depicted (**c** & **f**) with white arrows highlighting the presence of intracellular ThT-reactive amyloid (green). Magnification X 1000, scale bars: 20 µm.

Spectral fluorescence profiles through modifications to existing fluorescence filter cubes demonstrate the isolation of lumogallion and ThT fluorescence signals.

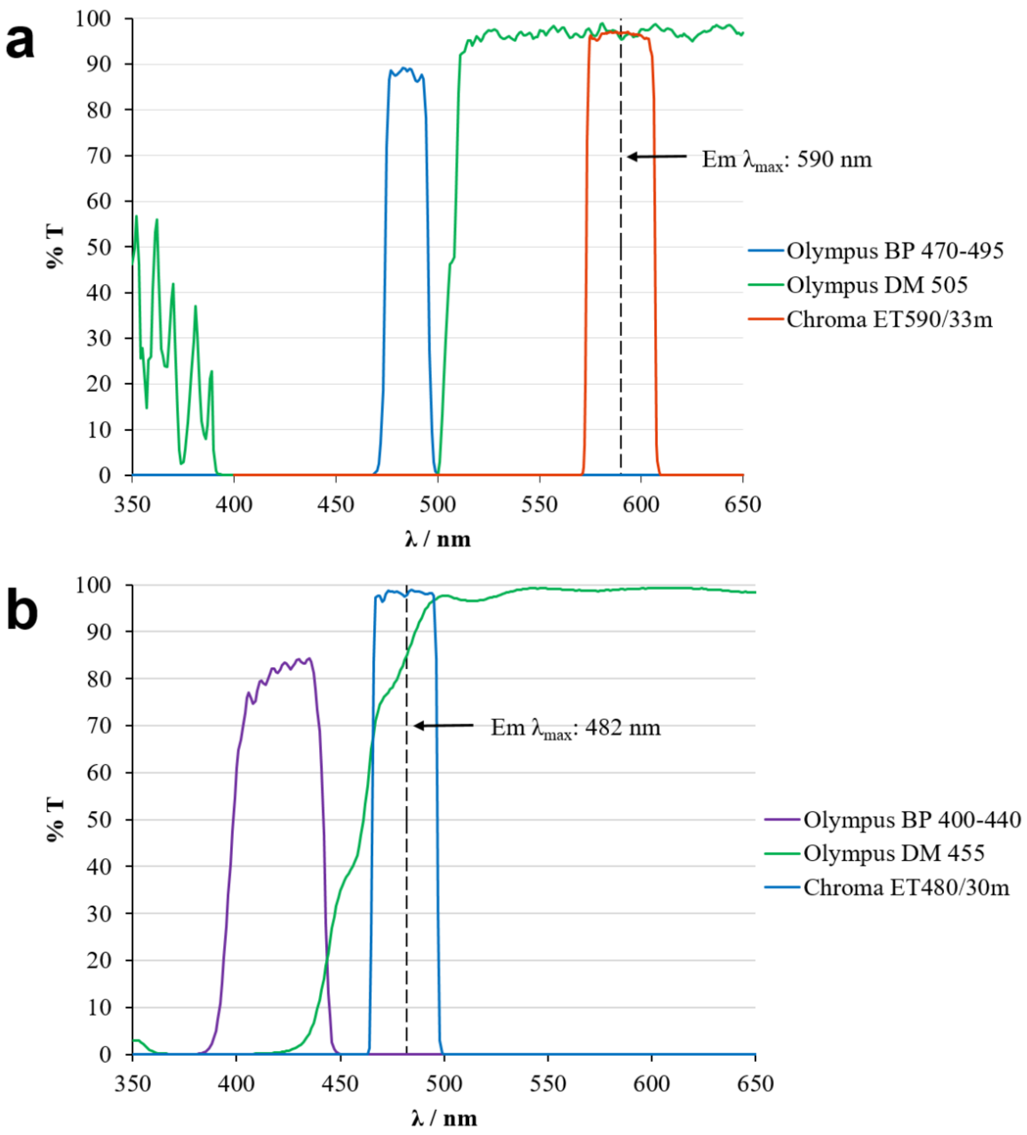

Supplementary Figure 5 Manufacturers measured spectral profiles of modified single bandpass fluorescence filters used in the visualisation of lumogallion (**a**) and ThT (**b**) fluorescence. Lumogallion fluorescence (**a**) was acquired using a U-MNIB3 fluorescence filter cube (Olympus, UK) with the longpass emission filter swapped for an ET590/33m (Chroma®, Vermont, US) filter (excitation: 470 – 495 nm, dichromatic mirror: 505 nm, emission: 570 – 610 nm). ThT fluorescence (**b**) was acquired through an Olympus U-MWBV2 cube equipped with a Chroma® ET480/30m singleband pass emission filter (excitation: 400 – 440 nm, dichroic mirror: 455 nm, emission: 470 – 500 nm). The emission maxima (Em λ_{max}) for both complexed fluorophores and the manufacturer's filter details are indicated on the respective spectra.

ThT staining of mature amyloid fibrils of Aβ_{42} produces green and blue fluorescence under long and short band pass filters, respectively.

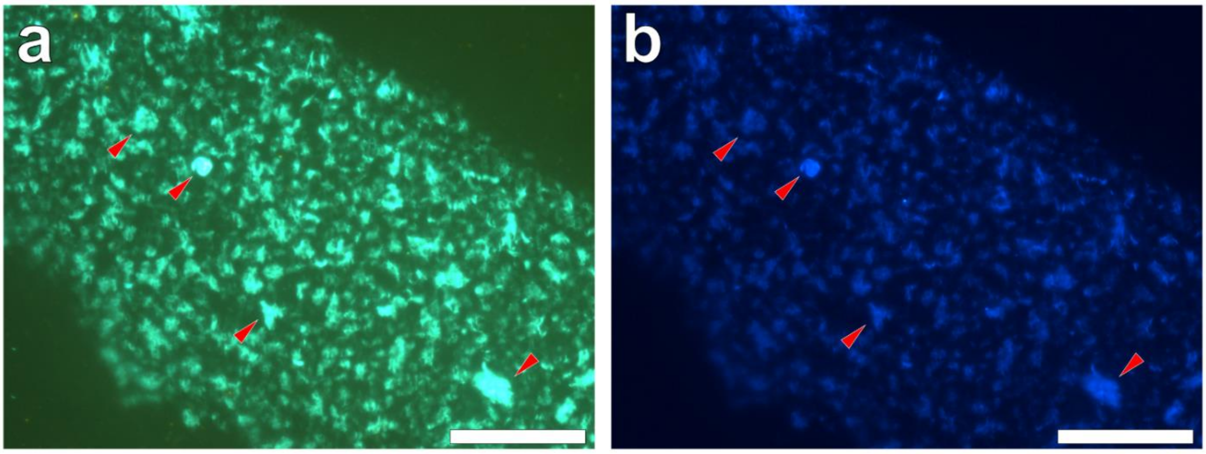

Supplementary Figure 6 Fluorescence microscopy of aged *ca* 29 µM Aβ_{42} stained in an agar support medium (5 µm) with 1 mM thioflavin T (ThT) for 24 h and viewed under long (**a**) and single (**b**) bandpass filters. ThT-reactive amyloid is identified as a green (**a**) versus a blue fluorescence (**b**) under long (≥ 510 nm emission) and single bandpass (Chroma® Vermont, US: 470 – 500 nm emission) filters housed in an Olympus U-MWBV2 filter cube, respectively. Arrows highlight ThT-reactive amyloid visible under both emission filters. Magnification X 400, scale bars: 50 µm.

ThT staining of THP-1 cells reveals ThT-reactive amyloid in only those cells co-cultured with a model Aβ$_{42}$ peptide antigen.

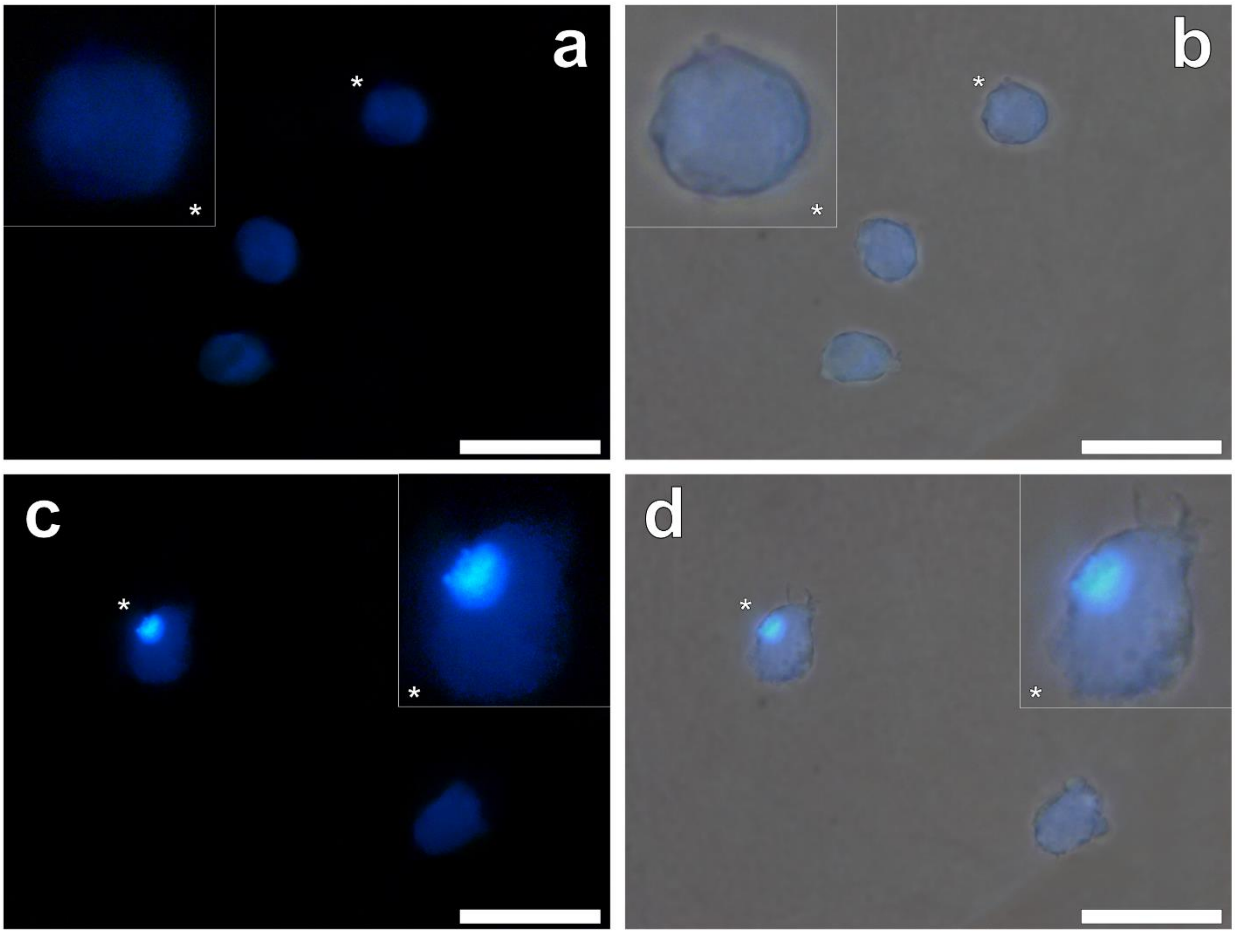

Supplementary Figure 7 Fluorescence micrographs and bright-field overlays of THP-1 cells co-cultured with *ca* 4 µM Aβ$_{42}$ in R10 medium in the absence (**a** & **b**) and presence (**c** & **d**) of *ca* 10 µM ThT, viewed under a modified U-MWBV2 single bandpass emission filter (excitation: 400 – 440 nm, dichromatic mirror: 455 nm, emission: 470 – 500 nm). Fibril formation of the peptide was initiated over 24 h at 37°C in R10 medium, prior to plating 1:1 with cells (final volume *ca* 200 µL per well). Following 21 h (37°C, 5% CO_2), ThT was diluted from a 1 mM stock solution (in ultrapure water) directly into plated cells at a final concentration of *ca* 10 µM. A further 3 h incubation in the absence (**a** & **b**) or presence (**c** & **d**) of the dye was performed, prior to fixation and mounting of whole cells onto poly-lysine coated slides. Magnified inserts of whole cells are denoted (*) revealing ThT fluorescence (blue) confined to intracellular compartments. Magnification X 1000, scale bars: 20 µm.

Supplementary methods

Preparation of R10 cell culture media

All chemicals were purchased from Sigma Aldrich, UK unless otherwise stated. Complete R10 cell culture medium was prepared via the addition of thawed 10% *v/v* heat-inactivated foetal bovine serum (certified US origin) to RPMI 1640 containing GlutaMAX™ supplement and 25 mM HEPES (both purchased from Fisher Scientific, Invitrogen). Gentamicin, an antimicrobial, was subsequently added from a 10 mg/mL stock solution in ultrapure water to a final concentration of 100 μg/mL. Complete media were stored at 4°C prior to use.

THP-1 cell culture

T helper 1 (THP-1) cells were purchased from ATCC (TIB-202, LGC Standards, UK) and were stored in the vapour phase of liquid nitrogen prior to use. Cell stocks were prepared at 1 x 10^6 cells / mL in R10 medium containing 5% *v/v* dimethyl sulfoxide (DMSO). Cells were thawed rapidly and diluted tenfold using warm R10 medium. In order to remove the DMSO, cells were subsequently centrifuged at low speed (10 min, 200 *g*) and upon removal of the supernatant, cells were re-suspended in warm R10 medium. Cells were initially transferred into tissue culture (TC)-treated vented and canted T25 culture flasks (VWR, Corning®, UK) and allowed to proliferate in a dedicated cell incubator, under humidified conditions at 37°C containing 5% atmospheric CO_2. Once cells had reached a density of 1 x 10^6/mL as counted by use of a haemocytometer, subcultures were continued in T75 flasks.

Assessment of amyloid fibril formation in R10 cell culture medium

The formation of amyloid fibrils was assessed in treatments containing $A\beta_{42}$ by use of a modified benzothiazole dye based ThT assay[25]. ThT was prepared at *ca* 11 µM in modified Krebs Henseleit medium containing 118.5 mM NaCl, 4.8 mM KCl, 1.2 mM $MgSO_4$, 1.4 mM $CaCl_2$ and 11.0 mM glucose buffered in 100mM PIPES at pH 7.4, including 0.05% *w/v* NaN_3 to inhibit microbial growth. ThT fluorescence was quantified using an LS55 fluorimeter equipped with a PTP-1 fluorescence Peltier system (Perkin Elmer, UK).

Background fluorescence intensity (fl_c) was measured at an excitation wavelength of 450 nm over 300 s, in which 90 µL ThT was added to a low volume quartz cell maintained at 37°C (excitation: 450 nm: 5 nm bandpass, emission: 482 nm: 10 nm bandpass). Ten µL of the given treatment was added such that the final ThT concentration was *ca* 10µM. Following a 60 s incubation period at 37°C in the heated sample chamber, the fluorescence was measured as before (fl_t). The final fluorescence reading was defined as $fl_f = fl_t - fl_c$ [25].

Preparation of poly-lysine slides for fluorescence imaging

Following incubation with the treatment, cells were aspirated from wells and pooled into respective treatment conditions, in 1.5 mL microfuge tubes (Eppendorf, UK). To each tube, 400 µL of R10 was added, prior to centrifugation for 10 min at 200 *g*, to pellet the cells. Following removal of the supernatant, cells were reconstituted in 4% *w/v* paraformaldehyde (PFA) in 150 mM NaCl and 25 mM PIPES buffered at pH 7.4. Cells were fixed via incubation at ambient temperature for 20 min, after which cells were pelleted via

centrifugation for 10 min at 4000 *g*. Cells were re-suspended in a PIPES based buffer (150 mM NaCl, 50 mM PIPES, pH 7.4) and the process was twice repeated to wash cells of residual PFA. Cells were finally re-suspended in 200 μL of the same PIPES buffer ready for addition to poly-lysine coated slides.

Using a PAP pen for immunostaining, a hydrophobic square barrier of 1.5 cm by 1.5 cm, was drawn on to POLYSINE® slides (Thermo Scientific, UK). Fixed and washed cell suspensions were added to poly-lysine slides in humidity chambers, to prevent evaporation during adherence. Following a 20 min incubation period at ambient temperature, excess liquid was drawn away from a single corner of the hydrophobic square and discarded. Slides were subsequently mounted with an aqueous Fluoromount™ mounting medium and stored horizontally away from light at 4ºC, prior to imaging via fluorescence microscopy.

Spurr-resin embedding of THP-1 cells for transmission electron microscopy

THP-1 cells prepared under the varying culture regimes were embedded into Spurr resin to allow for subsequent sectioning for TEM as previously described[27]. PFA fixed cells were pre-embedded into an agar support medium (5% *w/v*) in BEEM® capsules, to prevent the loss of cells during processing. The blocks were first dehydrated through an ethanol gradient series from 30% to 100% *v/v*. Spurr resin was prepared via sequential addition of 13g NSA (nonenyl succinic anhydride, R1054), 5g ERL 4221 (3,4-epoxycyclohexylmethyl 3,4-epoxycyclohexyl carboxylate), 3g DER (diglycidyl ether of polypropylene glycol, R1074) and 0.2g S-1 (dimethylaminoethanol, R1067) (Elektron Technology, Agar Scientific, UK). Resin components were combined via slow mixing for a maximum of 15 min.

Gradual infiltration of Spurr resin was achieved by transferring dehydrated blocks into 3:1 dry 100% *v/v* ethanol : Spurr resin for 2 h, 1:1 dry ethanol : Spurr resin for 2 h and 1:3 dry ethanol : Spurr resin overnight (16 h). Three changes of fresh undiluted Spurr resin were implemented over 8 h to allow for the resin to fully infiltrate the blocks. Resin-embedded blocks were arranged into moulds and polymerisation was performed using a resin oven maintained at a temperature of 60°C, for a minimum of 16 h.
